# Mismatch between subjective and objective dysautonomia

**DOI:** 10.1038/s41598-024-52368-x

**Published:** 2024-01-30

**Authors:** Peter Novak, David M. Systrom, Sadie P. Marciano, Alexandra Knief, Donna Felsenstein, Matthew P. Giannetti, Matthew J. Hamilton, Jennifer Nicoloro-SantaBarbara, Tara V. Saco, Mariana Castells, Khosro Farhad, David M. Pilgrim, William J. Mullally

**Affiliations:** 1 Autonomic Laboratory, Department of Neurology, Brigham and Women’s Hospital, Boston, MA USA; 2 Department of Neurology, Brigham and Women’s Faulkner Hospital, Boston, MA USA; 3 Harvard Medical School, Boston, MA USA; 4 Department of Medicine, Pulmonary and Critical Care, Brigham and Women’s Hospital, Boston, MA USA; 5 Department of Infectious Disease and Medicine, Massachusetts General Hospital, Boston, MA USA; 6 Department of Medicine, Mastocytosis Center, Brigham and Women’s Hospital, Boston, MA USA; 7 Department of Psychiatry, Brigham and Women’s Hospital, Boston, MA USA; 8 Department of Neurology, Massachusetts General Hospital, Boston, MA USA

**Keywords:** Neuroscience, Physiology

## Abstract

Autonomic symptom questionnaires are frequently used to assess dysautonomia. It is unknown whether subjective dysautonomia obtained from autonomic questionnaires correlates with objective dysautonomia measured by quantitative autonomic testing. The objective of our study was to determine correlations between subjective and objective measures of dysautonomia. This was a retrospective cross-sectional study conducted at Brigham and Women’s Faulkner Hospital Autonomic Laboratory between 2017 and 2023 evaluating the patients who completed autonomic testing. Analyses included validated autonomic questionnaires [Survey of Autonomic Symptoms (SAS), Composite Autonomic Symptom Score 31 (Compass-31)] and standardized autonomic tests (Valsalva maneuver, deep breathing, sudomotor, and tilt test). The autonomic testing results were graded by a Quantitative scale for grading of cardiovascular reflexes, sudomotor tests and skin biopsies (QASAT), and Composite Autonomic Severity Score (CASS). Autonomic testing, QASAT, CASS, and SAS were obtained in 2627 patients, and Compass-31 in 564 patients. The correlation was strong between subjective instruments (SAS vs. Compass-31, r = 0.74, *p* < 0.001) and between objective instruments (QASAT vs. CASS, r = 0.81, *p* < 0.001). There were no correlations between SAS and QASAT nor between Compass-31 and CASS. There continued to be no correlations between subjective and objective instruments for selected diagnoses (post-acute sequelae of COVID-19, n = 61; postural tachycardia syndrome, 211; peripheral autonomic neuropathy, 463; myalgic encephalomyelitis/chronic fatigue syndrome, 95; preload failure, 120; post-treatment Lyme disease syndrome, 163; hypermobile Ehlers-Danlos syndrome, 213; neurogenic orthostatic hypotension, 86; diabetes type II, 71, mast cell activation syndrome, 172; hereditary alpha tryptasemia, 45). The lack of correlation between subjective and objective instruments highlights the limitations of the commonly used questionnaires with some patients overestimating and some underestimating true autonomic deficit. The diagnosis-independent subjective–objective mismatch further signifies the unmet need for reliable screening surveys. Patients who overestimate the symptom burden may represent a population with idiosyncratic autonomic-like symptomatology, which needs further study. At this time, the use of autonomic questionnaires as a replacement of autonomic testing cannot be recommended.

## Introduction

Dysautonomia either primary or in association with other diseases, is common, affecting millions of patients worldwide. The exact number is unknown but it is estimated that postural tachycardia syndrome (POTS), a subset of dysautonomia, affects 1–3 million patients in the United States^1^.

An objective diagnosis of dysautonomia using quantitative autonomic testing has limited availability in the US and worldwide due to a shortage of autonomic testing centers. To circumvent this shortcoming, questionnaire surveys have been increasingly used for the assessment of dysautonomia as a surrogate for objective assessments of autonomic dysfunction. Commonly used questionnaires are the Composite Autonomic Symptom Score 31 (Compass)-31^2^ and Survey of Autonomic Symptoms (SAS)^3^. The advantage of questionnaires include little to no cost, they are easy to administer, and do not require a specialized autonomic center. Nevertheless, symptoms are a poor predictor of dysautonomia^4,5^, and the lack of correlations or poor correlations between autonomic questionnaires and autonomic testing was already noted in previous smaller studies^3,6,7^. For example, the SAS validation study showed no association between the SAS and Composite Autonomic Severity Score (CASS)^8^, a validated instrument for grading autonomic failure using cardiovascular reflex and sudomotor tests, or CASS subscores^3^. The authors concluded that early diabetic neuropathy is associated with mild autonomic neuropathy that current autonomic tests are not sensitive enough to detect or that autonomic symptom scores overrate for the presence of autonomic neuropathy.

Compass-31 was derived from the COMPASS, an 84-question scoring instrument, which was derived from the Autonomic Symptom Profile (ASP), a 169-items instrument^9^. The COMPASS score correlated with CASS (r = 0.69); however, in the subsequent diabetic study, ASP correlated poorly with CASS for type 1 diabetes (r = 0.37 for secretomotor and urinary domains, no total score was given) but correlations were absent for the orthostatic intolerance domain and for all domains in type 2 diabetes^7^.

Correlation studies between self-report dysautonomia questionnaires and objective assessments of autonomic dysfunction in a larger cohort are lacking. This study aimed to evaluate how subjective dysautonomia as assessed by validated autonomic questionnaires correlates with objective dysautonomia as assessed by standardized autonomic testing in a large cohort of patients.

## Materials and methods

The study was approved by the Institutional Review Board (IRB) of the Brigham and Women’s Hospital, Harvard Medical School, as a minimal-risk study. IRB has waived the need for informed consent for this study. All research was performed in accordance with relevant guidelines and regulations.

### Participants

This retrospective, single-center study included consecutive patients who underwent autonomic testing between 2017 and 2023 at the Brigham and Women’s Faulkner Hospital Autonomic Laboratory for evaluation of dysautonomia. Patients’ electronic records were reviewed to obtain details about past medical history, laboratory evaluations, and medication.

The patient’s diagnoses were obtained from medical records. The diagnoses of POTS, neurogenic orthostatic hypotension, and neurally mediated syncope were determined by autonomic testing. The diagnosis of peripheral autonomic neuropathy (PAN)^10,11^ was obtained from the medical records and confirmed by abnormal autonomic testing. In general, PAN patients have a combination of symptoms of dysautonomia affecting cardiovascular, gastrointestinal, urogenital, and sudomotor systems and abnormal functional autonomic testing. We also required for PAN diagnosis to have abnormal skin biopsies at the legs for assessment of epidermal and sudomotor autonomic fibers, thus satisfying criteria for mixed small fiber neuropathy^12^. In this study, diabetic neuropathy is reported as a separate diagnosis.

Orthostatic intolerance was defined as the presence of chronic (≥ 6 months) symptoms of cerebral hypoperfusion including lightheadedness, dizziness, shortness of breath, palpitations, brain fog, fatigue, and blurred vision with standing and relief of symptoms with recumbency. POTS was defined as the presence of symptomatic orthostatic intolerance along with an increment in heart rate ≥ 30 BPM of duration > 1 min during the tilt test without orthostatic hypotension^13^. Neurogenic orthostatic hypotension was defined as a decline in blood pressure by 20/10 mmHg of systolic/diastolic blood pressure compared to supine baseline and reduced the compensatory orthostatic heart rate increase^14^.

Exclusion criteria included the use of medication that affect autonomic functions during autonomic testing for duration less than 5 half-lives. The excluded medication were antihistamines, antidepressants, antihypertensives, beta-blockers, anticholinergics, acetylcholinesterase inhibitors, drugs for orthostatic hypotension, phenothiazines, asthma inhalers except for steroid inhalers. We also required to abstain from nicotine and caffeine for at least 3 h and from alcohol for at least 24 h before the study. Patients experiencing syncope during the testing were also excluded.

### Subjective dysautonomia assessments

To increase the power of our study, we used two validated subjective instruments: SAS^3^ and Compass-31^2^. SAS was designed as an instrument to measure autonomic symptoms in mild dysautonomia as seen in early diabetic neuropathy. The SAS is a self-administered 12-item self-report questionnaire (11 items in women, 12 items in men) that assesses autonomic symptom severity over the past 6 months over six domains (orthostatic, sudomotor, vasomotor, gastrointestinal, urinary, and sexual). The severity of each item is rated from 1 (least severe) to 5 (most severe), the SAS range is 0–55 for women and 0–60 for men. Compass-31 was proposed as a general instrument for quantitative measures of autonomic symptoms and function. Compass-31 is a 31-item inventory that assesses autonomic symptom severity over six autonomic domains (orthostatic, vasomotor, secretomotor, gastrointestinal, bladder, and pupillomotor) over the last year. Scores range from 0 to 100 with higher scores indicating more severe symptoms. SAS was filled by most of the patients a few days before autonomic testing. Compass-31 was filled by patients on the day of testing.

### Autonomic tests

All testing was performed following established standards and has been previously described in details^15^. Briefly, cardiovascular reflex tests included deep breathing, the Valsalva maneuver, and the tilt test. Patients were tilted at 70° for 10 min following 10 min of supine rest. The tilt was aborted if the subject developed syncope. Sudomotor assessment was done using electrochemical skin conductance (ESC)^16^.

Recorded signals included electrocardiogram, blood pressure, respiratory movement, end-tidal CO_2_, respiratory rate, and CBFv in the middle cerebral artery using Transcranial Doppler. Blood pressure was obtained intermittently using an automated oscillometric sphygmomanometer Welch Allyn CVSM 6400 Monitor (Skaneateles Falls, NY) from the arm and continuously using Finometer® (Finapress Medical Systems, Amsterdam, Netherlands) from the thumb or index finger. All signals were recorded using PowerLab 16/35 data acquisition system with LabChart 8 software (ADInstruments Inc., Colorado Springs, CO, USA) and sampled at 400 Hz.

There are two validated instruments for grading autonomic test results: CASS^8^ and Quantitative scale for grading of cardiovascular reflex, sudomotor tests, and skin biopsies (QASAT)^17^. The CASS is a 11-point scoring instrument of autonomic failure. The scale rates sympathetic adrenergic deficits (0–4) and sympathetic sudomotor and parasympathetic cardiovagal deficits (both 0–3). This total score ranges from 0 (no deficit) to 10 (maximal deficit). QASAT originated from CASS and was validated in 612 patients with variety of neurological disorders. In addition to dysautonomia, QASAT grades small fiber neuropathy and cerebral blood flow abnormalities.

QASAT’s additional domains such as cerebral blood flow and skin biopsies, will not be reported in this study. Autonomic failure subscore of QASAT have been shown to strongly correlate with CASS (r = 0.84, *p* < 0.0001)^17^.

Deep breathing test is an establised test for assessment of parasympathetic cardiovagal functions^8^ and is used in both QASAT and CASS. QASAT parasympathetic score was obtained from the QASAT heart rate variability score which is identical to CASS parasympathetic score. Blood pressure responses to Valsalva maneuver and tilt test are established tests for evaluation of sympathetic adrenergic functions^8,18^. Both QASAT and CASS use Valsalva maneuver and tilt test but the main difference is the calculation of tilt responses. While both instruments use the supine position for baseline measurements, CASS asseses blood pressure responses at 1, 5, and 10th minute of the tilt, while the QASAT uses blood pressure responses at every minute of the tilt. An additional difference is the definition of orthostatic hypotension during the tilt. CASS uses 30/20 mmHg of the systolic/mean orthostatic blood pressure decline while QASAT uses systolic/diastolic orthostatic blood pressure decline measured either in percent (20%/10%) or in mmHg (20 mmHg/10 mmHg), the latter is recommended by a consensus^19^. In this study we used the 20 mmHg/10 mmHg criterion. QASAT sympathetic score is defined as summation of QASAT Valsalva maneuver in blood pressure score and QASAT orthostatic hypotension score. Compared to CASS, the dynamic range is 0–13 for QASAT sympathetic adrenergic score and 0–4 for CASS-sympathetic adrenergic score.

The sudomotor test is used for sudomotor scoring in both CASS and QASAT. CASS uses Quantitative Sudomotor Axon Reflex Test (QSART)^8^ while QASAT allows choice between QSART and Electrochemical skin conductance (ESC)^16^. For the purpose of this study we used ESC for several reasons: (1) There is a shortage of acetylcholine in the United States needed for QSART; (2) QSART might overestimate the sudomotor deficit^20^; (3) QSART has limited test–retest reliability^21,22^; (4) QSART correlates poorly with skin biopsies assessing small fibers^17^; (5) ESC correlate with skin biopsies and ESC has modest accuracy to detect loss of sudomotor fibers^16^. QASAT score grading autonomic failure (QASAT_af_) was defined by^23^:

$${\text{QASAT}}_{{{\text{af}}}} = {\text{QASAT}}_{{{\text{parasympathetic}}}} + {\text{QASAT}}_{{{\text{sympathetic}}}} + {\text{QASAT}}_{{{\text{sudomotor}}}} .$$

Composite CASS score for grading of autonomic failure was defined by^8^:

$${\text{CASS}} = {\text{CASS}}_{{{\text{parasympathetic}}}} + {\text{CASS}}_{{{\text{sympathetic}}}} + {\text{CASS}}_{{{\text{sudomotor}}}}$$

CASS scores were calculated using the algorithm published previously^24^. QASAT version 0.2 scores were calculated using the the qpack package written in python (www.python.org)^23^.

### Statistical analysis

Since the data did not have a normal distribution, Spearman rank correlation coefficients were calculated for continuous variables. Patients with missing data were excluded from analysis. R statistical software (www.r-project.org) with package ggstatsplot^25^ was used for statistical analyses.

## Results

**Figure 1:**
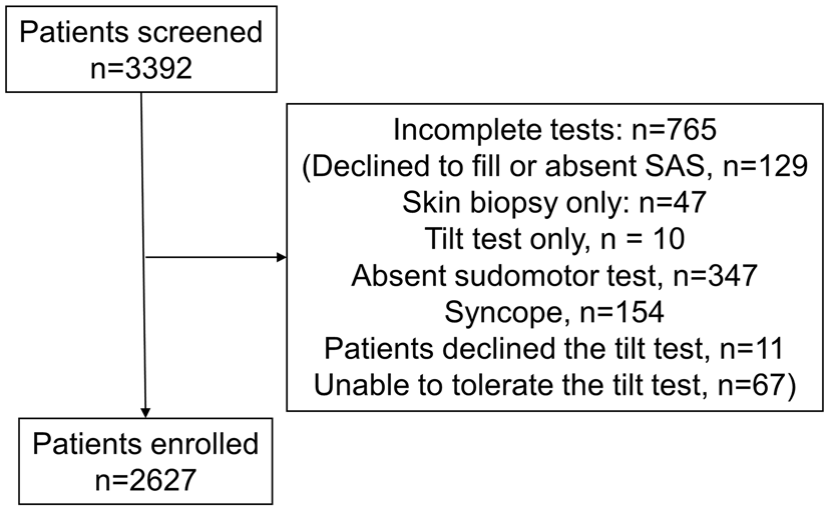
STROBE diagram showing flow of the study.

A total of 3392 patients were referred for autonomic testing. From them, 2627 completed autonomic tests and were included in this study (Fig. 1). Autonomic tests, QASAT, CASS, and SAS were available in 2627 patients, while Compass-31 was obtained in 564 out of 2627 patients since we added COMPASS-31 to our evaluations in 2022.

**Figure 2:**
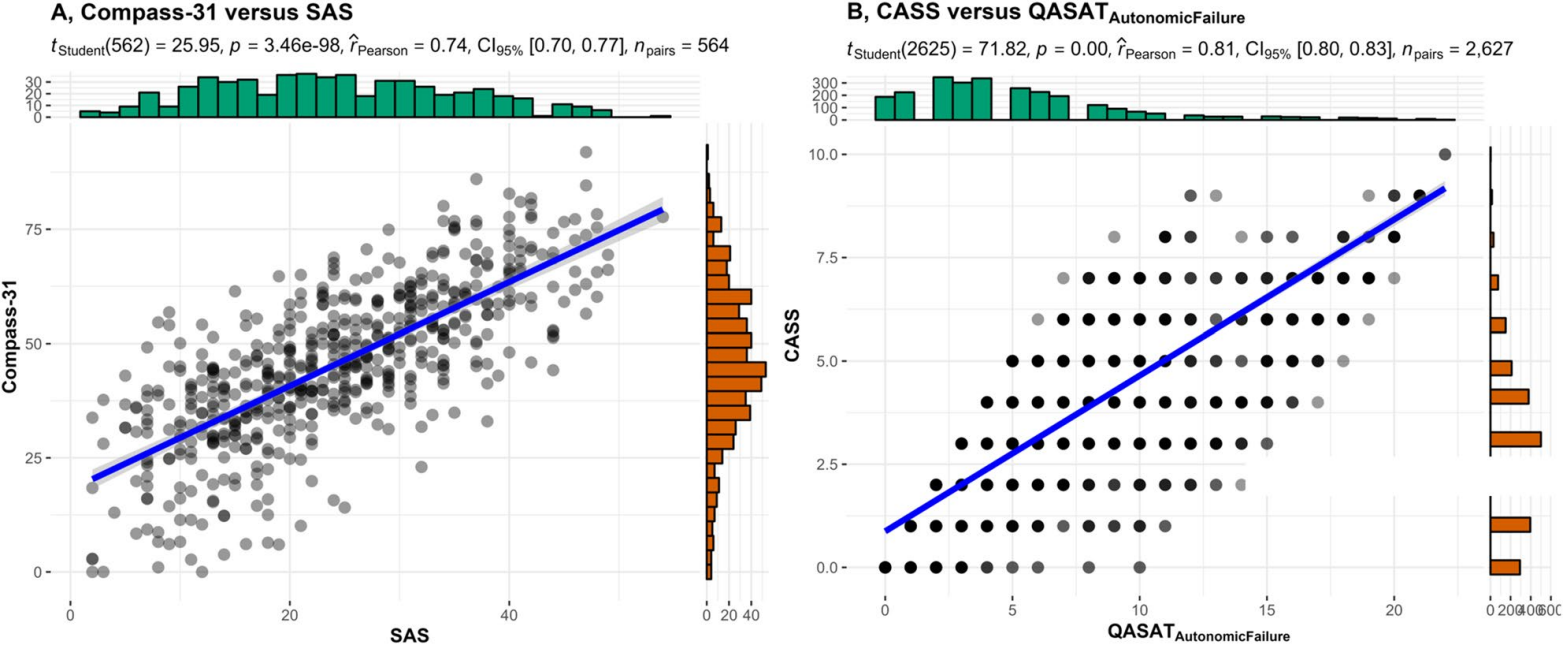
Correlations between Compass-31 versus SAS (**A**) and CASS versus QASAT_af_ (**B**).

**Figure 3:**
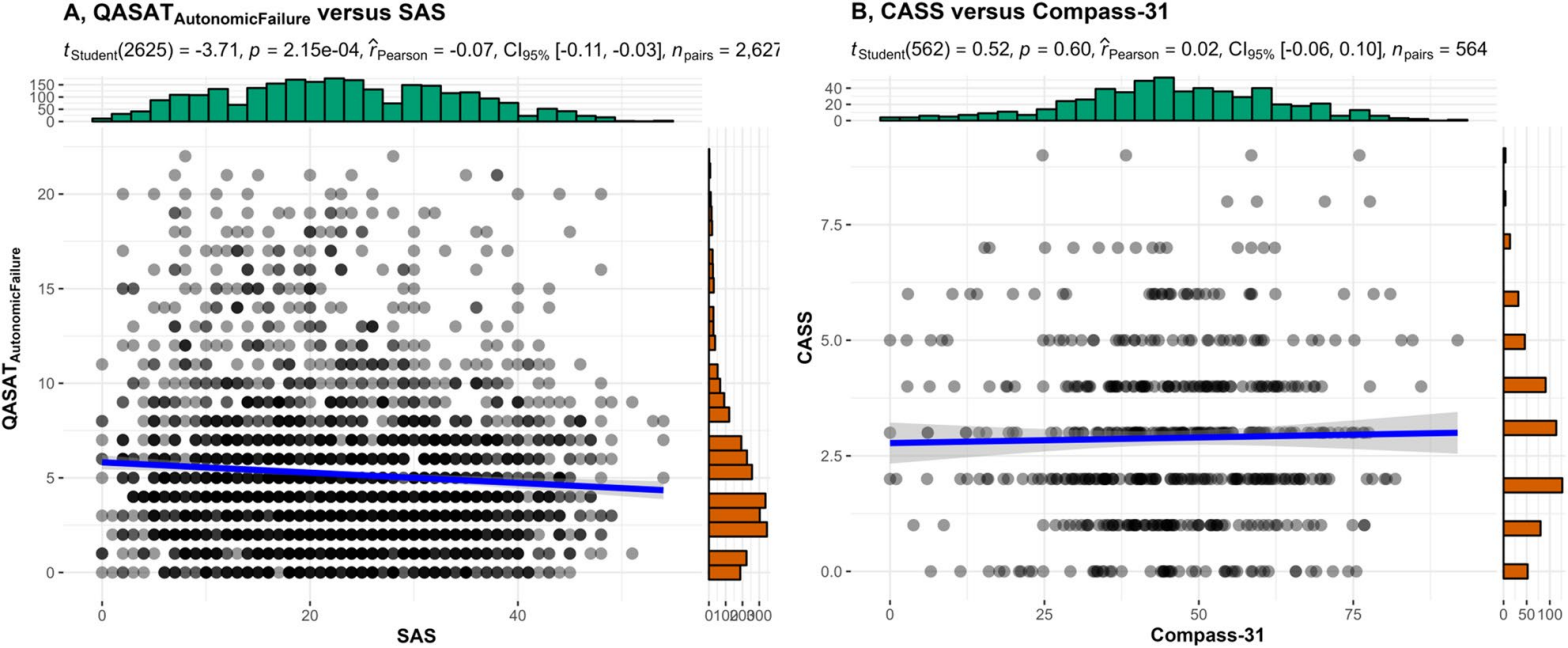
Absent correlations between QASAT_af_ versus SAS (**A**) and between CASS versus Compass-31 (**B**).

Correlations were strong between subjective instruments (SAS vs. Compass-31, r = 0.74, *p* < 0.001, Fig. 2A) and between objective instruments (QASAT vs. CASS, r = 0.81,* p* < 0.001, Fig. 2B). There was no correlation between SAS and QASAT_af_ (Fig. 3A). There was no correlation between Compass-31 and CASS (Fig. 3B). Correlations between subjective and objective instruments remained absent for selected diagnoses (post-acute sequelae of COVID-19, POTS, hypermobile Ehlers-Danlos syndrome, peripheral autonomic neuropathy, preload failure, myalgic encephalomyelitis/chronic fatigue syndrome, post-treatment Lyme disease syndrome, neurogenic orthostatic hypotension, diabetes type II, mast cell activation syndrome, and hereditary alpha tryptasemia confirmed by genetic testing Table 1).

**Table 1:** Correlations between symptoms and testing scores for all subjects and most common diagnoses.

Symptoms	Signs	All subjects n = 2627	PASC n = 61	POTS n = 211	PAN n = 463	ME/CFS n = 95	PF n = 120	PTLDS n = 163	NOH n = 86	DM n = 71	EDS n = 213	MCAS n = 172	HAT n = 45
SAS	QASAT-AutonomicFailure	− 0.06, 0.001	− 0.01, 0.962	0.05, 0.455	− 0.02, 0.65	− 0.09, 0.404	0, 0.988	− 0.04, 0.598	− 0.01, 0.9	− 0.07, 0.537	0.06, 0.38	0.08, 0.313	− 0.09, 0.55
	QASAT-Sympathetic	− 0.04, 0.068	− 0.01, 0.923	0.01, 0.853	0, 0.917	− 0.16, 0.132	− 0.07, 0.435	0, 0.995	− 0.01, 0.93	− 0.16, 0.193	− 0.01, 0.852	0.09, 0.223	0.05, 0.751
	QASAT-Parasympathetic	− 0.02, 0.363	− 0.13, 0.326	− 0.01, 0.895	0.01, 0.856	− 0.11, 0.3	− 0.02, 0.786	0.06, 0.431	− 0.04, 0.597	0.05, 0.678	0.09, 0.194	0.11, 0.163	− 0.06, 0.708
SAS-Orthostatic	QASAT-AutonomicFailure	− 0.08, 1e−04	0, 0.991	0.11, 0.096	− 0.05, 0.265	0.06, 0.537	0, 0.988	− 0.04, 0.598	− 0.01, 0.9	− 0.07, 0.537	0.06, 0.38	0.08, 0.313	− 0.09, 0.55
	QASAT-sympathetic	− 0.04, 0.049	0.09, 0.512	0.12, 0.08	− 0.03, 0.493	0.09, 0.386	− 0.07, 0.435	0, 0.995	− 0.01, 0.93	− 0.16, 0.193	− 0.01, 0.852	0.09, 0.223	0.05, 0.751

At each line, the top number is the rho (correlation coefficient) and the bottom number is the p (level of significance).

SAS, Survey of Autonomic Symptoms; QASAT, Quantitative scale for grading of cardiovascular reflexes; sudomotor tests and skin biopsies; PASC, post-acute sequale of COVID-19; POTS, postural tachycardia syndrome; PAN, peripheral autonomic neuropathy; ME/CFS, myalgic encephalomyelitis/chronic fatigue syndrome; PF, preload failure; PTLDS, post-treatment Lyme disease syndrome; NOH, neurogenic orthostatic hypotension; EDS, hypermobile Ehlers-Danlos syndrome; DM, diabetes; type 2; MCAS, mast cell activation syndrome; HAT, hereditary alpha tryptasemia.

## Discussion

The main finding of this study is the lack of correlation between subjective dysautonomia (assessed by SAS and Compass-31) and objective dysautonomia (assessed by CASS and QASAT_af_). On the other hand, strong correlations were found between subjective instruments (SAS vs. Compass-31) and between objective instruments (CASS vs. QASAT_af_).

There was good agreement between subjective instruments (SAS vs. Compass-31) even though Compass-31 and SAS were designed to to assess different scenarios. Compass-31 was designed to asses autonomic complaints in patients with dysautonomia. SAS was designed to detect mild dysautonomia in patients with diabetes. SAS strongly correlates with Compass-31 when applying to diabetic as well as non diabetic patients indicating that both instruments are comparable and capture similar information. SAS can be a reliable alternative to Compass-31 as the simplicity and shorter length of SAS may offer reduced patients burden and easier administration.

Strong correlation between objective autonomic instruments (CASS vs. QASAT) indicate that both instruments agree in determination of severity of autonomic failure. This finding is not surprising as CASS is a subset of QASAT, the main difference is the expanded dynamic range of QASAT for sympathetic adrenergic scoring. Neither dysautonomia questionaire correlates with objective assessments of autonomic dysfunction. It can be argued that questionnaires have wider scope as they assess domains that are not assessed by autonomic testing such as the gastrointestinal or urinary/bladder domains. However even orthostatic complaints, a subset of subjective dysautonomia, did not correlate with variety of objective dysautonomia subscores including sympathetic adrenergic score that grades the blood pressure responses to orthostatic stress.

The lack of correlation between subjective and objective instruments provides insight into the limitations of the self repored tools with some patients overestimating symptoms and some underestimating true autonomic deficit. The subjective–objective mismatch was not diagnosis-dependent as the lack of correlations was noted in disorders associated with dysautonomia such as neurogenic orthostatic hypotension, small fiber neuropathy, preload failure, and diabetes type II, as well as in disorders where the dysautonomia is not well defined. The latter category includes myalgic encephalomyelitis/chronic fatigue syndrome, post-treatment Lyme disease syndrome, and mast cell disorders. The diagnosis-independent subjective–objective mismatch, however, further highlights the unmet needs of autonomic questionnaires. Patients who overestimate the symptom burden may represents a population with idiosyncratic dysautonomia, which needs further study.

The results confirm previous findings^3,6,7^ that patient-reported autonomic symptoms are a poor predictor of objective autonomic dysfunction. Obviously other factors contributing to subjective dysautonomia should be considered such as cerebral hypoperfusion associated with hypocapnic cerebral hypoperfusion^26^ and orthostatic cerebral hypoperfusion syndrome^27^ as well as comorbidities including depression, anxiety, stress, fatigue, central sensitization^28–32^, and mast cell disorders^33^. Clinical features that are associated with subjective but not objective dysautonomia may be better characterized as a separate entity and studied in future studies.

### Limitations

Referral bias and single-center cross-sectional retrospective character constitue main limitations of this study. However the study sample is large and therefore the results may be generalized to at least US patients. Ideally, the study should be replicated for the non-US subjects. There exist other patient-reported instruments evaluating autonomic symptoms that were not used in our study. However, these instruments are not applicable for generalized evaluations of dysautonomia since they are focusing on a specific autonomic features. For example, the orthostatic hypotension questionnaire was designed to assess the symptoms of neurogenic orthostatic hypotension^34^ while the new Malmo POTS score was design to assess the autonomic symptoms severity in POTS^35^. It is also possible that the association between subjective and objective instruments is confounded by other variables and it may be uncovered by calculating partial correlations. Finally, the dependency between subjective and objective dysautonomia can be non-linear and its detection may require to use of more specialized methods.

## Conclusions

Our study showed that there is no correlation between subjective dysautonomia determined by questionaires and objective dysautonomia determined by quantitative autonomic testing. Future studies are needed to determine the origin and clinical significance of patient-reported subjective dysautonomia. At this time, the use of autonomic questionnaires as a replacement of autonomic testing cannot be recommended.

## Data Availability

Data are available from P.N. upon reasonable request.
